# Editorial Brevities

**Published:** 1888-02

**Authors:** 


### EDITORIALS AND MISCELLANEOUS.

### EDITORIAL BREVITIES.

COCAINE.—It is said that much of the cocaine now in the market is adulterated with borax.

THE GEORGIA MEDICAL ASSOCIATION meets in Rome, on the third Wednesday in April next. The chairman of the committee of arrangements is Dr. Robt. Battey we learn.

DR. HARDMAN, of Monroe, Georgia, an esteemed physician, and well remembered as the Surgeon of the Forty-second Georgia Regiment, in the Confederate service, died recently at his home in Monroe, Ga.

MIDDLETON GOLDSMITH, M. D., formerly Professor in the Kentucky School of Medicine died on 26th ultimo, at Rutland, Vermont. Dr. Goldsmith was noted on the staff of General Grant.

DEATH OF DR. PALMER.—We note the death of A. B. Palmer, M. D., Professor of Practice in the University of Michigan. He died at Ann Arbor, on December 23, 1887, aged seventy-two years.

ANATOMICAL MATERIAL.—Our medical colleges report favorably of the workings of our new law relating to the ptotection of cemeteries and the furnishing of material for the use of the schools in the study of anatomy.

A SANITARY CONFERENCE is soon to be held at Lima, in Peru. Surgeon-General Hamilton has been deputized by our Government to attend this conference. Perhaps we may learn something more of the yellow fever germs, and the method of preventing the malady.

☞ When a practitioner concludes to economize by cutting off one of his medical journals, let it not be his home journal. It is the duty of every true man in the profession to sustain his home journal, and to aid in promoting the medical litterature and the advancement of medical science in his own section.

WE acknowledge a New Year's greeting from William R. Warner & Co., "that staunch and reliable" house doing business both in Philadelphia and New York. Their New Year's Card is gotten up in beautiful and tasteful style. We warmly reciprocate the friendly token.

They have a new and interesting advertisement in this issue of our journal, which do not fail to examine.

WHITE'S PHYSIOLOGICAL MANIKIN.—Don't fail to see the advertisement in this issue of White's manikin—very useful to the student and practitioner, and a splendid office ornament. We will furnish one of these manikins to any one who will send us thirty-five subscribers to the RECORD. Medical societies would do well to secure one of these manikins by clubbing together and sending us the required number of subscribers.

WHOOPING COUGH HOLDS ITS OWN.—It is often asked can whooping-cough be cut short. We have seen many cases this winter, and after trying nearly all of the vaunted remedies found in the RECORD of late years, we have to admit that the disease ran its usual course with only slight palliation in some cases.

SCARLATINA AND THE COW.—It is now claimed by Prof. Crookshank, of King's College, London, that the disease in the cow which Klein declared to give rise to scarlatina in man from the use of the milk is none other than true Jennerian cow-pox. It would thus appear that there is a close relation between scarlatina and small pox.

WE had the pleasure recently of meeting with Dr. T. R. WHITLY, of Douglasville, Georgia. He gives a favorable account of the Sweet Water Medical Association, of which he is the Secretary. The Association is in flourishing condition, and is made up of a large representation of medical gentlemen from a number of counties. The meetings are held quarterly at Austell, Ga., to-wit, the first Wednesday in January, April, July and October.

THE SOUTHERN MEDICAL COLLEGE COMMENCEMENT will take place at DeGives' Opera House, in this city, on the evening of 29th of February next. The Annual Address will be delivered by Colonel NISBET, of Atlanta, who will also deliver the prizes.

The exercises of the Dental Department will take place at the same time. The occasion is looked forward to as one of much interest and pleasure. The members of the profession and citizens generally are cordially invited to be present.

THANK YOU, DOCTOR.—We receive from many of our subscribers kind and complimentary words, which greatly encourage us in our arduous labors. To publish all these, however pleasing it might be to our vanity, would scarcely be proper, and would take up too much of our space. But I must quote a sentence from a letter just received, enclosing a remittance from Dr. R. L. BRANCH, an esteemed subscriber of South Carolina, in which he says of THE RECORD: "It is too good a journal to read without paying for in advance." We trust that all of our friends will take due notice thereof and govern themselves accordingly.

